# 3D bioprinting using a new photo-crosslinking method for muscle tissue restoration

**DOI:** 10.1038/s41536-023-00292-5

**Published:** 2023-03-31

**Authors:** JaeYoon Lee, Hyeongjin Lee, Eun-Ju Jin, Dongryeol Ryu, Geun Hyung Kim

**Affiliations:** 1 Department of Precision Medicine, Sungkyunkwan University School of Medicine, Suwon, Republic of Korea; 2 Department of Biotechnology and Bioinformatics, Korea University, Sejong, Republic of Korea; 3 Department of Molecular Cell Biology, Sungkyunkwan University School of Medicine, Suwon, Republic of Korea; 4 Department of Biomedical Science and Engineering, Gwangju Institute of Science and Technology, Gwangju, Republic of Korea; 5 Department of Biophysics, Institute of Quantum Biophysics, Sungkyunkwan University, Suwon, Republic of Korea

**Keywords:** Tissue engineering, Translational research

## Abstract

Three-dimensional (3D) bioprinting is a highly effective technique for fabricating cell-loaded constructs in tissue engineering. However, the versatility of fabricating precise and complex cell-loaded hydrogels is limited owing to the poor crosslinking ability of cell-containing hydrogels. Herein, we propose an optic-fiber-assisted bioprinting (OAB) process to efficiently crosslink methacrylated hydrogels. By selecting appropriate processing conditions for the photo-crosslinking technique, we fabricated biofunctional cell-laden structures including methacrylated gelatin (Gelma), collagen, and decellularized extracellular matrix. To apply the method to skeletal muscle regeneration, cell-laden Gelma constructs were processed with a functional nozzle having a topographical cue and an OAB process that could induce a uniaxial alignment of C2C12 and human adipose stem cells (hASCs). Significantly higher degrees of cell alignment and myogenic activities in the cell-laden Gelma structure were observed compared with those in the cell construct that was printed using a conventional crosslinking method. Moreover, an in vivo regenerative potential was observed in volumetric muscle defects in a mouse model. The hASC-laden construct significantly induced greater muscle regeneration than the cell construct without topographical cues. Based on the results, the newly designed bioprinting process can prove to be highly effective in fabricating biofunctional cell-laden constructs for various tissue engineering applications.

## Introduction

Recently, cell-loaded scaffolds mimicking natural complex tissue structures have been fabricated using a variety of bottom-up methods, such as electrohydrodynamic, microfluidic, photo- or soft-lithographic, and three-dimensional (3D) bioprinting processes^1–5^. Owing to the efficient production ability demonstrated by multi-layer techniques, the 3D bioprinting process has been widely adopted in tissue engineering applications. Particularly, versatile applications of various bioinks have been significantly explored^6–9^.

Previously, many promising results have been made in the development of tissue-engineered skeletal muscle with improved functionalities without bioprinting^10,11^. However, a notable disadvantage of tissue-engineered skeletal muscle constructs without bioprinting is the inadequacy to fabricate patient-specific geometries. To overcome this issue, various parameters including printing temperature, pneumatic pressure, printing speed, and the crosslinking process to fabricate 3D cell constructs with appropriate biological activities, such as high cell viability/proliferation and phenotypic differentiation/maturation of the printed cells should be considered.

However, nozzle-based extrusion processes demonstrate several shortcomings, such as limited strut-size resolution, restrained cell-density of cell-loaded struts, and low printability, which resulted from the weak mechanical nature of cell-loading hydrogels. Several advanced bioinks conjugated with ions have been introduced to obtain cell constructs; however, the fabrication of mechanically stable cell-loaded structures using the 3D printing technology remains a challenge to overcome in the printing technique and bioink formation process^8,12,13^.

Recently, to overcome the shortcomings of cell-laden struts obtained using a 3D printing process, several crosslinking strategies, including in situ photo-crosslinking processes combined with printing, have been proposed^8,14,15^. For instance, fabricating mechanically stable microscale cell-loaded filaments requires printing processes to employ a core/shell nozzle system, wherein the alginate bioink in the shell region is immediately crosslinked with a calcium chloride solution that protects the photo-crosslinked cell-laden methacrylated gelatin (Gelma) in the core^16,17^. Additionally, similar studies have fabricated cell-loaded core (Gelma)/shell (sodium alginate) fibers using a microfluidic channel in which endothelial cell-laden Gelma in the core is designed^18^. In addition, an in situ crosslinking method using a transparent capillary coaxial nozzle connected to a 3D printer has been introduced to obtain stable printed structures (microscale lattices and hollow tubes) without depending on the bioink viscosity^8^.

The proposed printing processes have clearly demonstrated advanced results in obtaining cell-laden 3D microfibrous structures that mimic the intrinsic morphologies and biofunctions of real fibrous tissues; however, the methods employ limited bioink (i.e., use of rapid crosslinkable alginate in calcium ions) to support cell-loaded bioactive materials. Furthermore, for in situ photo-crosslinkable systems, a realistic multilayered 3D structure cannot be easily obtained owing to the barely photo-crosslinked shell region of the flowing methacrylated cell-laden hydrogels, and a specifically transparent and hydrophobic nozzle is required.

To address these challenges encountered by previous printing systems, we present a new photo-crosslinking strategy combined with a printing process to fabricate mechanically stable structures and realistic multilayered cell-loaded structures. To that end, we used an optical fiber embedded within a general printing nozzle to stably photo-crosslink flowing methacrylated hydrogels (methacrylated gelatin, collagen, and decellularized extracellular matrix (dECM)). Using this unique crosslinking system, mechanically stable and biologically safe cell-laden struts and a multilayered 3D cell-laden structure constructed with reasonable adhesive force between cell-loaded filaments were effectively fabricated. To apply the versatility of the photo-crosslinking process, surface-micropatterned cell-laden struts, using C2C12 and human adipose stem cells (hASCs) processed with a surface-grooved plastic nozzle, were fabricated for muscle tissue regeneration. The cell constructs with a uniaxially grooved surface pattern demonstrated enhanced myogenic differentiation owing to cell alignment achieved by the combination of the grooved surface in the nozzle and in situ optic-fiber-assisted crosslinking process. Furthermore, the bioprinted hASC constructs were applied in a volumetric muscle defect injury in mice, and they demonstrated significantly enhanced muscle regeneration compared with those fabricated using a conventional bioprinting process.

## Results and discussion

### Photo-crosslinking of the 3D bioprinting process supported with an optical fiber

In general, photo-crosslinkable bioinks, such as methacrylated hydrogels based on gelatin, collagen, hyaluronic acid, and decellularized extracellular matrix, have been widely used in fabricating cell-loaded constructs owing to their outstanding cell-encapsulating abilities and controllable mechanical properties that can be effectively achieved using various photo-crosslinking processes^9,19–22^. When photo-crosslinkable bioinks are combined with a general bioprinting process, multilayered cell-laden constructs with customizable pore geometries can be fabricated successfully. However, the precise and stable formation of cell constructs fabricated using bioprinting supplemented with a photo-crosslinking process critically depends on the crosslinking approach employed. Of the various crosslinking strategies that are combined with bioprinting, the most breakthrough strategy is the “in situ photo-crosslinking method” executed using a transparent hydrophobic silicone nozzle, which can lower the shear stress of a flowing photo-crosslinked-bioink^8^. When the bioink passes through the nozzle, UV light can be directly exposed to the transparent nozzle to crosslink the flowing photo-crosslinkable and low-viscosity bioink. This method is of immense significance in that it effectively and stably crosslinks low-viscosity bioinks to attain mechanically stable cell-laden struts. However, the in situ photo-crosslinking process requires a specially designed transparent and hydrophobic photo-permeable nozzle. Furthermore, because the surface of the cell-laden struts can be more actively crosslinked than the core region of the printed struts, the adhesive property between the struts can be relatively poor after fabricating 3D cell structures, resulting in a low 3D structural shape-ability.

**Fig. 1:**
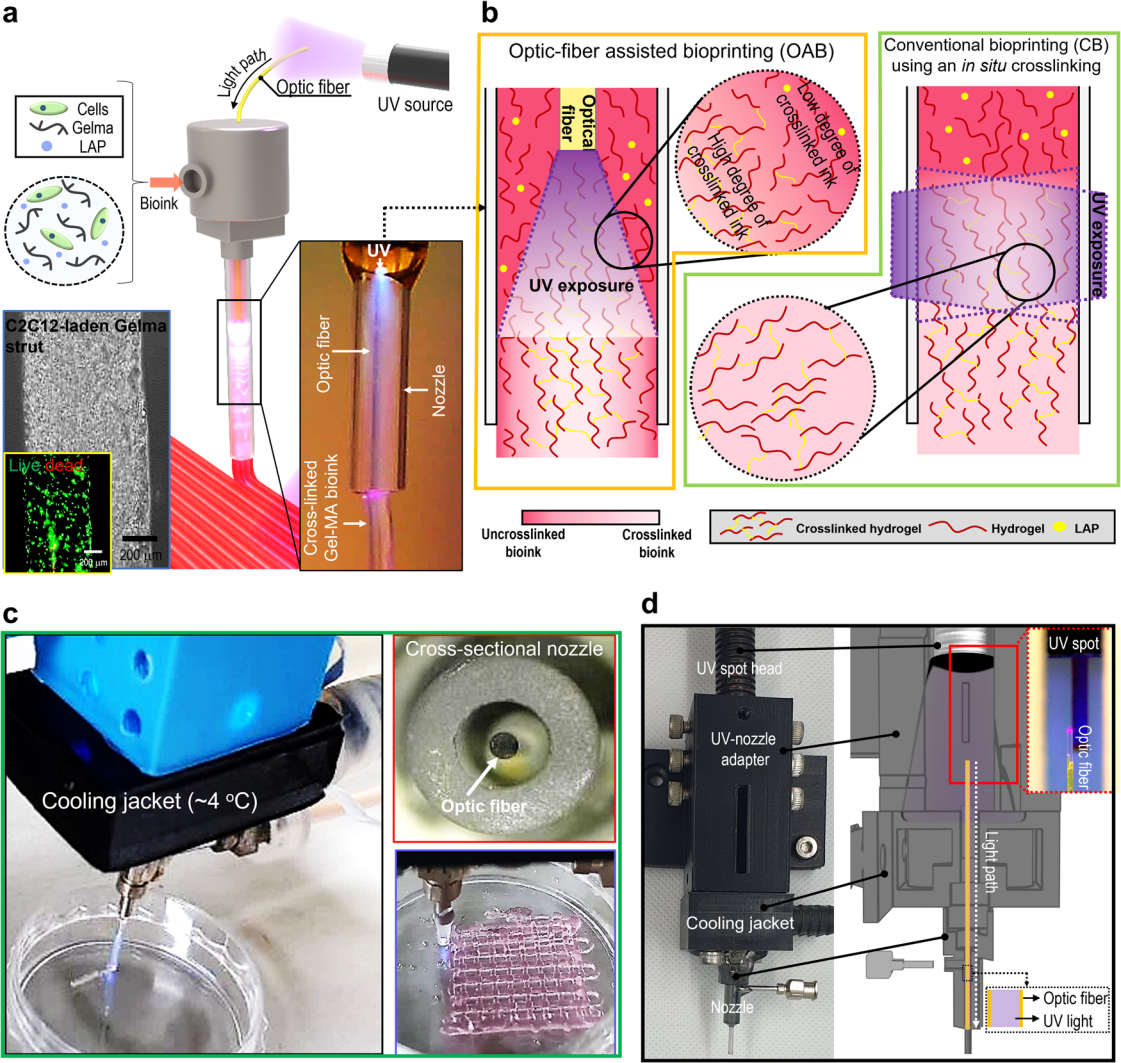
Schematics of an optic-assisted bioprinting process. **a** Schematic of an optic-assisted bioprinting process (OAB) using an optical fiber placed within a nozzle of a printer, and optical and live (green)/dead (red) images of the printed C2C12-laden Gelma strut. **b** Schematics of a photo-crosslinked hydrogel within a nozzle processed with OAB and CB process. **c**, **d** OAB system connected with a water-cooling jacket, cross-sectional printing nozzle image, and an optical image of a printed mesh cell-laden Gelma structure.

To overcome the shortcomings of in situ crosslinking of photo-crosslinkable bioinks, we developed a new crosslinking method using an optic-fiber-assisted 3D bioprinting (OAB) process that was completely different a conventional bioprinting (CB) using an in situ crosslinking process (Fig. 1a–c). To assess the performance of the OAB process, we selected a photo-crosslinkable Gelma-based bioink laden with C2C12 myoblasts with a density of 1 × 10^7^ cells/mL. This is because Gelma can be considered a typical photo-crosslinkable bioink with biocompatible and biodegradable properties, and it can be widely applied in tissue engineering scaffolds, drug delivery systems, and healing substrates of wound regions^23–25^. To efficiently crosslink the bioink, an optical fiber was embedded in a general printing nozzle, and by controlling the intensity of a UV light source (UV-spot head, shown in Fig. 1d), we could obtain a mechanically stable and biologically safe 3D cell-laden construct, as presented in the optical and live (green)/dead (red) images in Fig. 1a and the optical image of the printed Gelma mesh-structure (Fig. 1c). Figure 1a, c presents an optical image of UV light as observed through the optical fiber inserted within the nozzle. In addition, to avoid a rise in the temperature of the bioink by the optical fiber, a cooling water jacket (4 °C) was set around the printing nozzle (Fig. 1c, d). Compared to conventional post-crosslinking, the proposed OAB process can obtain cell-laden 3D structures with intrinsic morphologies, as demonstrated by the enhanced circularity values (Supplementary Fig. 1). Additionally, the OAB method can fabricate a realistic multilayered 3D structure in contrast to CB, owing to enhanced strut-to-strut adhesion.

### Selection of appropriate crosslinking and printing conditions for the OAB process

**Fig. 2:**
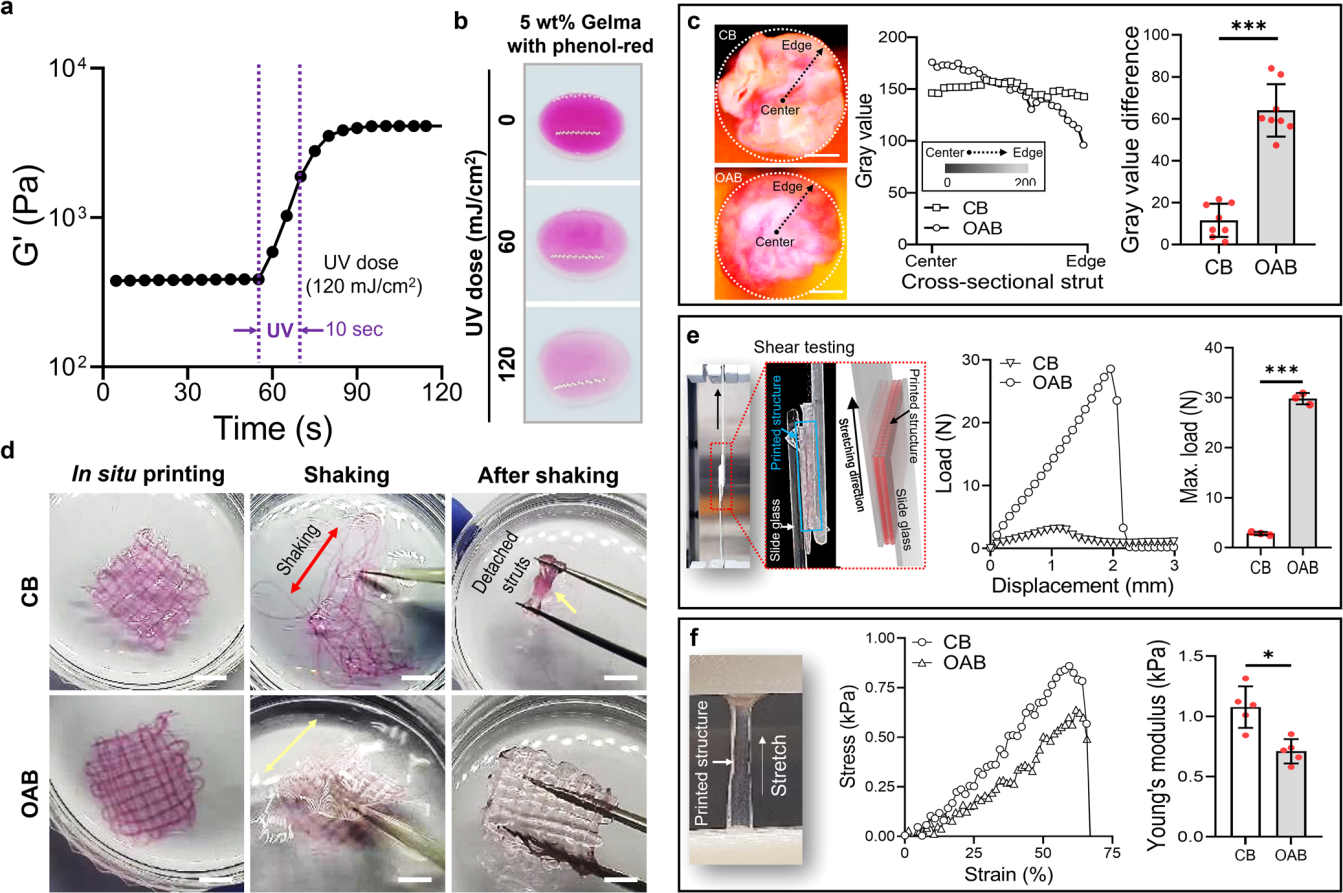
Physical characterizations of Gelma structure. **a** Storage modulus (G′) of 5 wt% Gelma obtained from time-sweep tests conducted before and after UV exposure (UV dose = 120 mJ/cm^2^ for 10 s). **b** Color change in the mixture of 5 wt% Gelma and phenol-red at various UV doses. **c** Cross-sectional optical images of the printed Gelma/phenol-red struts fabricated using conventional bioprinting (CB) and OAB processes, the distribution of the gray value using the cross-sectional images for radius, and the difference of gray value at the center and edge regions. **d** Optical images of printed Gelma mesh structures after in situ printing, during shaking, and after shaking to illustrate the structural stability. **e** Shear testing of the printed mesh structures processed with CB and OAB to observe the adhesive properties between the struts. **f** Tensile stress–strain curves and Young’s modulus of the printed Gelma structures. All data are presented as mean ± SD. The *p* values were calculated by student’s *t* test (*n* = 3/group; *p < 0.05; **p < 0.01; ***p < 0.001). Scale bar, 200 µm (**c**); 1 mm (**d**).

To observe the effect of the OAB process on the photo-crosslinking ability of the Gelma bioink, 5 wt% Gelma was used. Figure 2a illustrates the storage modulus (G′) of the Gelma bioink in the time-sweep mode before and after UV exposure (UV dose = 120 mJ/cm^2^ for 10 s). As expected, the stiffness of Gelma exposed to UV light was significantly enhanced after UV exposure.

To observe the effect of photo-crosslinking produced by the optical fiber placed in the interior of the nozzle on the crosslinking distribution over the cross-sectional area of a Gelma strut, we measured the distribution of the red-color intensity of phenol-red, which differed depending on the photo-crosslinking degree. Figure 2b depicts the various red-color intensities for the mixture of 5 wt% Gelma/phenol-red (150 mg/L) at different UV doses (0, 60, and 120 mJ/cm^2^), indicating that the red-color faded into white at an increasing UV dose. Figure 2c illustrates two cross-sectional images of Gelma struts that were crosslinked using the CB and OAB processes, respectively. In the printing/crosslinking processes, the size of the printing nozzle, nozzle speed, pneumatic pressure, and UV dose were 700 μm, 1 mm/s, 120 ± 10 kPa, and 120 mJ/cm^2^, respectively.

The distribution of the red color for the photo-crosslinked gelatin struts fabricated using the CB and OAB methods is illustrated in Fig. 2c. As indicated in the results, the cross-sectional distribution of gray color illustrating the degree of photo-crosslinking was different for each method. For the CB process, the relative gray color distribution in the cross-sectional region of the Gelma strut was similar at the radius, whereas for the Gelma strut processed with the OAB, photo-crosslinking was relatively higher in the core region than in the shell region (the graph of the gray value difference in Fig. 2c). From these results, we can expect that the relatively low degree of crosslinking in the shell region of the strut can affect the 3D structural formation of multilayered printed filaments. Figure 2d illustrates printed mesh structures fabricated using the CB and OAB processes (5 wt% Gelma, UV dose = 120 mJ/cm^2^, nozzle size = 700 μm, nozzle moving speed = 2 mm/s, and pneumatic pressure = 120 ± 10 kPa). After fabricating the Gelma structures, the printed structures were shaken. As shown in the optical images, the 3D mesh structure was destroyed in the Gelma struts fabricated using the CB process, whereas the Gelma mesh structure fabricated using the OAB process was stably sustained in its originally designed shape. This result indicates that the gelatin struts printed using the OAB process adhered well to each other to sustain the 3D structural shape.

To evaluate the adhesive force between the Gelma struts, we measured the displacement versus load for shear testing (Fig. 2e). Consequently, the adhesive energy between the Gelma struts and the maximum load of the interfacial force between the Gelma struts fabricated using the OAB process were significantly higher than those for the Gelma struts processed using the CB process. This indicated that the 3D structural formations consisting of multilayered struts printed with the OAB process can be much more efficiently obtained than those printed with the CB process. However, for the tensile stress–strain curves, the structure processed with CB exhibited a significantly higher Young’s modulus than the structure printed using OAB (Fig. 2f). We believe that the difference in the tensile modulus could have resulted from the different degrees of crosslinking homogeneity within the struts, as shown in Fig. 2c. For the Gelma struts crosslinked with the OAB process, the externally applied tensile stress could be highly concentrated on the relatively low crosslinked region of the struts, inducing a lower resistance of the tensile force compared with that for the homogeneously crosslinked Gelma struts processed with CB.

To determine the appropriate range of printing parameters (UV dose and processing temperature) under a fixed Gelma weight fraction (5 wt%) and printing conditions (nozzle size = 700 μm, nozzle moving speed = 1 mm/s, and pneumatic pressure = 120 ± 10 kPa), a single line test was performed.

**Fig. 3:**
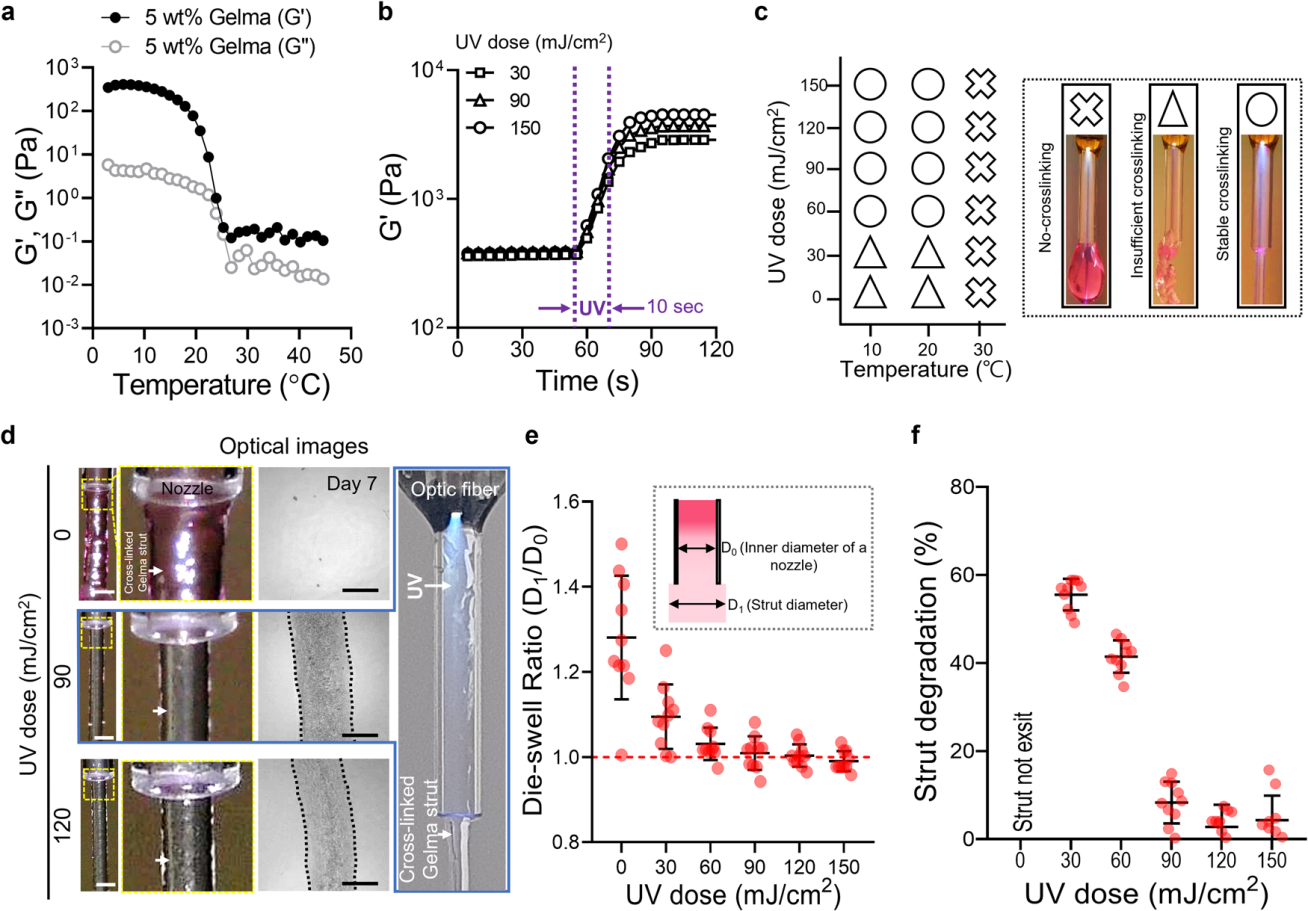
Photo-crosslinking ability of Gelma structures. Rheological properties of 5 wt% Gelma obtained via (**a**) temperature sweep and (**b**) time sweep at various UV doses. **c** Process diagram illustrating the effect of processing temperature and UV dose on the crosslinking degree of printed Gelma struts (no-crosslinking, insufficient crosslinking, and stable crosslinking). **d** Optical images of the printed shape of Gelma struts processed via OAB at various UV doses (0, 90, and 120 mJ/cm^2^) and optical images of the printed struts at 7 days of culture. **e** Die-swelling ratios (diameter of printed strut/diameter of the nozzle) for various UV doses. **f** Degradation of printed Gelma struts determined using the diameter difference observed before and after 7 days of culture. Scale bar, 1 mm (**d**—strut); 500 µm (**d**—cultured).

First, we measured the rheological properties of the 5 wt% Gelma bioink during temperature and time sweeps under various UV doses (30, 90, and 150 mJ/cm^2^). As expected, the Gelma bioink for the temperature sweep exhibited a typical sol–gel transition behavior (Fig. 3a). In addition, the increase in the UV dose at a constant temperature (10 °C) enhanced the crosslinking degree of the Gelma bioink (Fig. 3b).

To observe the effect of the temperature of the Gelma bioink on the photo-crosslinking degree, we selected three typical processing temperatures: (1) gel region: 10 °C, (2) gel–sol transition region: 20 °C, and (3) sol region: 30 °C for various UV doses (0–150 mJ/cm^2^). For the sol region temperature (30 °C), stable photo-crosslinking of the printed Gelma strut was not obtained regardless of the applied UV dose (0–150 mJ/cm^2^), whereas for Gelma struts printed at the gel and gel–sol transition temperatures and with more than 60 mJ/cm^2^ of UV doses, crosslinked Gelma struts were stably formed (Fig. 3c). The photo-crosslinking degree for various sol–gel temperatures of Gelma bioink was consistent with that reported in the study conducted by Chansoria et al.^26^. According to their research, the compressive modulus of Gelma bioink was highly dependent on not only the UV-exposure condition but also on the photo-crosslinking temperature; the modulus increased at lower crosslinking temperatures (gel temperature, 10 °C) of Gelma. This result was obtained because photo-crosslinking of the Gelma solution at low temperature could induce a synergistic effect of tightly entangled polypeptide networks at low temperatures and their photo-crosslinking by covalent binding^26^.

In addition, to demonstrate the structural stability after photo-crosslinking/printing, we measured the die-swelling ratio (D_1_/D_0_, where D_0_ and D_1_ indicate the diameter of the inner nozzle and strut diameter at the nozzle tip, respectively) and the change in the cross-sectional area of the Gelma struts after 7 days in the PBS solution. Figure 3d and Supplementary Fig. 2a depict optical images of the printed Gelma strut near the printing nozzle at a fixed printing temperature (10 °C) and various UV doses (0–120 mJ/cm^2^). Owing to the low crosslinking degree of Gelma exposed to UV doses of 0 and 30 mJ/cm^2^, the swelling ratio was significantly higher than that of the printed Gelma crosslinked at higher UV doses (90 and 150 mJ/cm^2^) (Fig. 3e). Additionally, the circularity of the extruded strut increased significantly until 120 mJ/cm^2^, however, the further increase in UV dose did not affect the circularity (Supplementary Fig. 2b). Furthermore, the flow velocity (Supplementary Fig. 2c, d) and optic fiber to nozzle outlet distance (Supplementary Fig. 2e, f) were investigated using a fixed UV dose (120 mJ/cm^2^). As result, the flow velocity had negligible effects on the circularity of extruded struts, whereas the increased distance between the optic fiber to nozzle distance had improved the circularity. A similar result was observed for the degradation behavior (=[A_0_ − A_1_]/A_0_, where A_0_ and A_1_ denote the cross-sectional areas of in situ printed struts and the area of the strut at 7 days, respectively) of the Gelma struts (Fig. 3f). For UV doses below 90 mJ/cm^2^, strut degradation was extremely rapid owing to insufficient crosslinking degrees. Based on the results obtained using strut formation and dimensions, we could select appropriate settings for the UV dose (120 mJ/cm^2^).

After fixing UV dose, we investigated various water jacket temperatures (4, 10, and 20 °C), as shown in Supplementary Fig. 3a, b. As a result, the homogeneity of the extruded strut measured using circularity and surface smoothness showed that the strut processed with the relatively low water jacket temperature (4 °C) indicated meaningfully higher circularity and smoother surface compared to those of the other temperatures. This can be attributed to the simultaneous UV crosslinking of Gelma at lower temperatures can induce expressive structural stability^27,28^. In addition, when the temperature of the water jacket is set at 4 °C, the printing temperature was measured to be 10 °C. After fixing the UV dose and printing temperature, the effect of the weight fraction of Gelma on the crosslinking degree was observed. Supplementary Figure 4a, b illustrates the storage modulus for the three weight fractions (3, 5, and 7 wt.%) of the Gelma bioink. As expected, the Gelma bioinks demonstrated a similar sol–gel transition for the temperature sweep, and crosslinking under a fixed UV dose (120 mJ/cm^2^) and printing temperature (10 °C) was achieved. After employing the UV- and fixed processing conditions, we recorded the optical images and measured the die-swelling ratio and degradation of the Gelma struts printed using the OAB process (Supplementary Fig. 4c–e). For all concentrations of the Gelma bioink, a die-swelling ratio of approximately 1 was observed; however, insufficient crosslinking was observed on the 3 wt% Gelma bioink (Supplementary Fig. 4e). Based on the results, we used a Gelma concentration of 5 wt% and the following printing parameters: UV dose = 120 mJ/cm^2^ and printing temperature = 10 °C for further evaluation. As shown in Supplementary Fig. 5, the dispersion of UV light inside the nozzle using the set parameters can evoke crosslinking in the core region of the extruded strut, whereas the shell region is crosslinked relatively less. Based on these data, we carefully predict that the optimized parameters in the OAB system can allow the fabrication of multilayered 3D structures.

### Application of the OAB process to obtain various structures

**Fig. 4:**
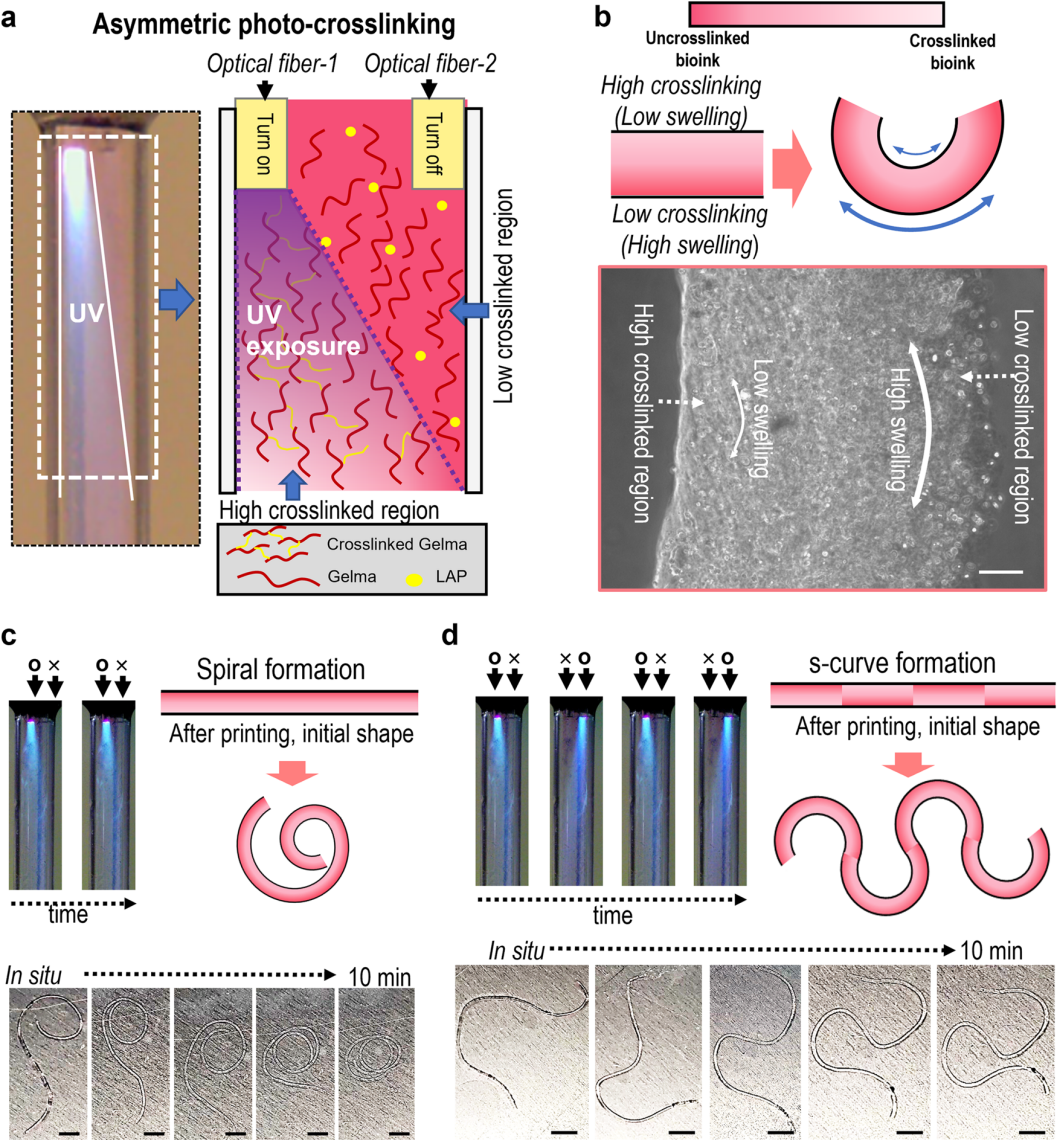
Shape-morphing Gelma Struts. **a** Optical and schematic of asymmetric photo-crosslinking using two optical fibers. **b** Shape-morphing mechanism of the Gelma strut and an optical image of the fabricated Gelma strut. Curving ability (**c** spiral and **d** ‘s’-curve formation) of the Gelma struts depending on asymmetric crosslinking of Gelma struts. Scale bar, 100 µm (**b**); 5 mm (**c**, **d**).

Recently, shape-morphing hydrogels or 4D biofabrication systems have been gaining attention owing to the enabling of biomimicry and intricate microarchitecture formation^29–31^. To expand the OAB process to the 4D fabrication of various complex biological architectures, we attached two optical fibers (optical fiber-1 and optical fiber-2) in a nozzle, shown in the schematic image of Fig. 4a. In the image, as the optical fiber-1 was turned on, the asymmetric photo-crosslinking of the printed Gelma structure can be attained. In general, high elastic property (≈high crosslinking degree) induced in low water swelling^32^, so that the degree of asymmetric crosslinking of the hydrogel seems to determine the direction of hydrogel curving. Figure 4b describes the curving mechanism in detail, and the optical image of the Gelma strut that was fabricated using the asymmetric photo-crosslinking process can indicate the asymmetric crosslinking of the strut. Figure 4c, d show the curving ability of the Gelma struts depending on the use of the optics fibers. When exposed to water, the asymmetrically photo-crosslinked Gelma struts initiated the different swelling rate, and diverse swelling behaviors occurred on the cross-sectional direction of the strut due to the different crosslinking degree, forming spiral and s-shaped structures. Based on the demonstrated proof-of-concept of the 4D fabrication, we carefully predict that the OAB system can be applied in various biological applications such as blood vessel formation or nerve grafts.

**Fig. 5:**
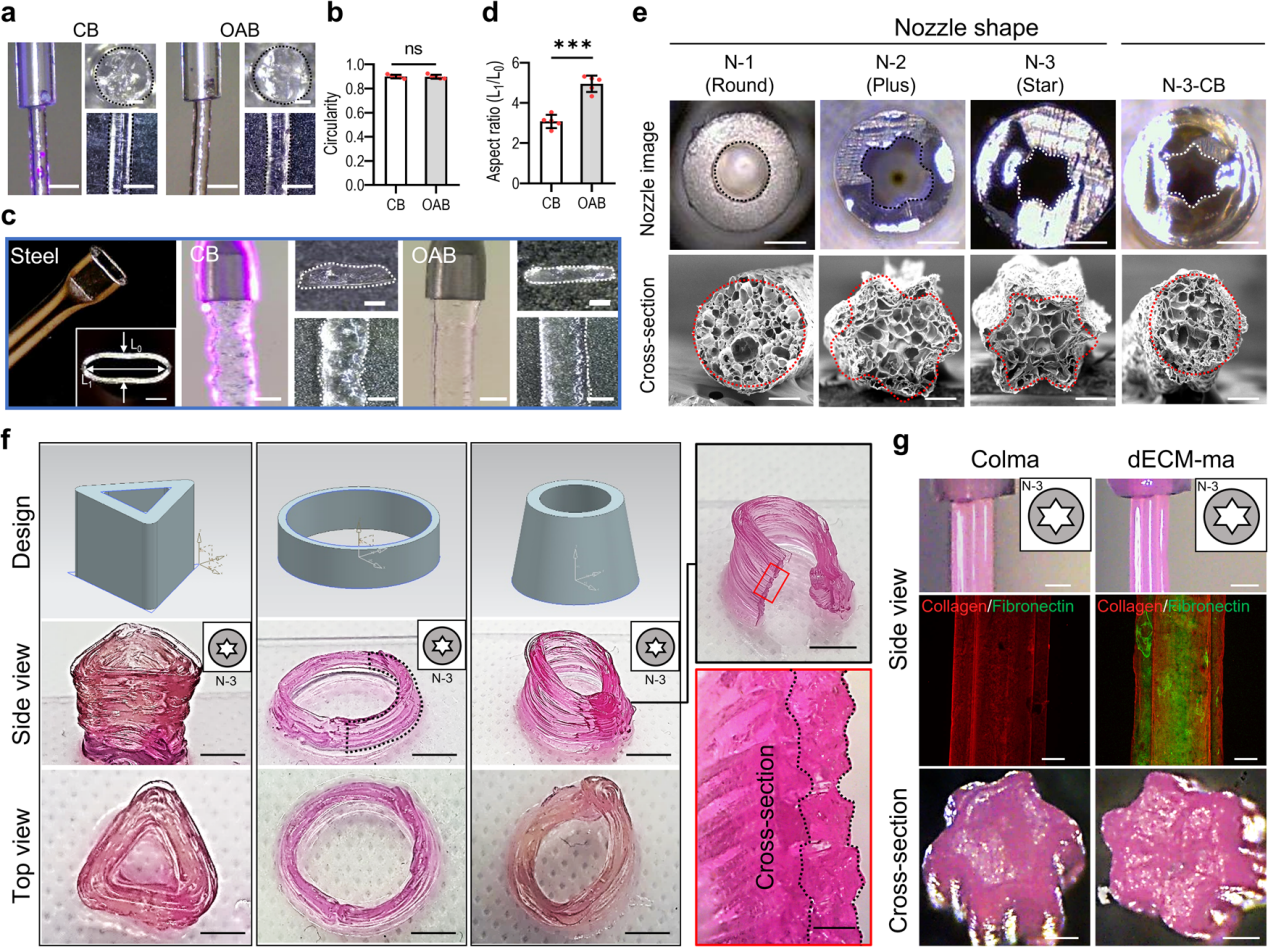
Formation of various shapes using OAB Processes. **a** Surface and cross-sectional optical images of the printed Gelma struts processed via CB and OAB (UV dose = 120 mJ/cm^2^) and **b** circularity of the cross-sectional images. **c**, **d** A steel nozzle with an ellipsoidal cross-sectional shape, printed Gelma struts processed via CB and OAB, and the aspect ratio (length of the long axis (L_1_)/length of the short axis (L_0_) of the printed strut) determined using the cross-sectional optical images of the printed struts. **e** Optical images of three plastic nozzles with different cross-sectional shapes (N-1: round, N-2: plus, N-3: star) and printed cross-sectional Gelma shapes fabricated using each plastic nozzle and the OAB process. N-3-CB indicates the cross-sectional optical image of Gelma processed with the plastic nozzle shape N-3 but crosslinked via the CB process, not the OAB process. **f** Optical images showing various Gelma 3D structures using the N-3 nozzle and OAB process and (**g**) optical and fluorescence (collagen: red and fibronectin: green) images of the surface and cross-sectional Colma and dECM-ma struts fabricated using the N-3 plastic nozzle and OAB process. All data are presented as mean ± SD. The *p* values were calculated by student’s *t*-test (*n* = 3/group; *p < 0.05; **p < 0.01; ***p < 0.001). Scale bar, 1 mm (**a**, **c**-nozzle and strut,); 200 µm (**a**, **c**—strut cross-section, **e**—SEM, **g**—fluorescence and cross-section); 500 µm (**c**, **e**—nozzle cross-section, **f**—cross-section, **g**—nozzle optical images); 5 mm (**f**—side view and top view).

As stated in the previous section, one of the advantages of the OAB process is that photo-crosslinkable bioinks can be crosslinked within the nozzle, regardless of the transparency of the nozzle material and nozzle complexity. Three different nozzles (glass nozzle, opaque plastic nozzle, and squeezed steel nozzle) were used to demonstrate the feasibility of the OAB process, independent of the specific nozzle design. As illustrated in Fig. 5a, a glass nozzle was used to extrude the Gelma bioink, and the Gelma struts printed with the CB and OAB processes under the previously set UV and printing conditions demonstrated a completely similar cylindrical shape (Fig. 5b). However, when the OAB process was employed using the ellipsoidal steel nozzle shown in Fig. 5c, a Gelma strut with a shape that was more similar to the shape of the steel cross-sectional nozzle than the strut shape printed with the CB process was obtained (Fig. 5c, d).

In addition, three opaque plastic nozzles with different cross-sectional shapes [round (N-1), plus-shaped (N-2), and star-shaped (N-3)] were used to print the Gelma bioink using the OAB process, as shown in Fig. 5e. The cross-sectional images of the Gelma struts printed with the OAB process exhibit shapes similar to those of the printing nozzles, whereas the Gelma strut (N-3-CB) printed with the N-3 nozzle and CB process does not exhibit a shape similar to that of the printing nozzle (cross-sectional star-shaped). Additionally, Supplementary Fig. 6 depicts optical images of printed Gelma strut with N-2 and N-3 nozzles at various UV doses (0–150 mJ/cm^2^). To show the 3D Gelma shapes fabricated using the OAB process and N-3 nozzle, we fabricated the shapes with three different structures, shown in Fig. 5f.

To expand the feasibility of the OAB process to different methacrylated hydrogels, a methacrylated collagen (Colma) (methacrylation degree: 79.3 ± 1.6%) and dECM methacrylate (dECM-ma, methacrylation degree: 76.0 ± 2.4%) that was derived from the skeletal muscle tissue were used. Schematic of decellularization/methacrylation processes using porcine muscle tissue was described (Supplementary Fig. 7a), and DNA contents and collagen, elastin, and GAG contents for the muscle tissue, Colma and dECM-ma were shown (Supplementary Fig. 7b–e). Figure 5g showed the surface and cross-sectional optical and fluorescence (collagen: red, fibronectin: green) images of the printed struts by using the N-3 nozzle and OAB process. The micro-grooved surface pattern was clearly observed in the Colma and dECM-ma struts.

### Application of the fabricated cell-laden struts to muscle tissue regeneration

**Fig. 6:**
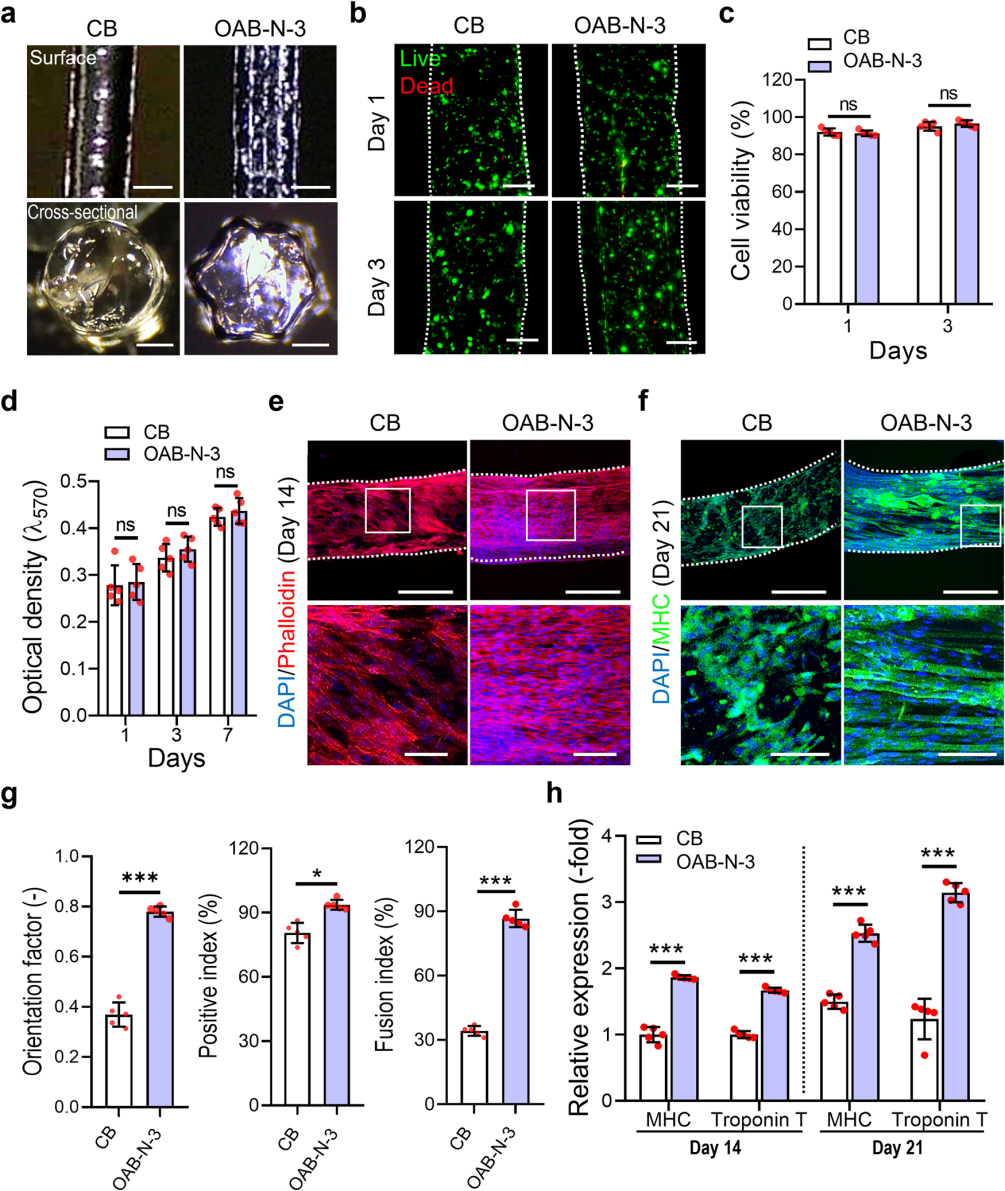
Comparison of in vitro cellular activities for the Gelma structures fabricated with CB and OAB processes. **a** Optical surface and cross-sectional images of the Gelma struts processed using CB and OAB processes with the N-3 nozzle. **b** Live (green)/dead (red) at 1 and 3 days of culture and (**c**) cell viability for each Gelma strut. **d** Cell proliferation determined using the MTT assay for the Gelma struts. **e** DAPI (blue)/phalloidin (red) at 14 days of culture and (**f**) DAPI/MHC (green) at 21 days of culture (white box indicate magnified region). **g** MHC orientation factor, positive index, and fusion index for the C2C12-laden Gelma struts at 21 days of cell culture. **h** Relative gene expression of MHC and Troponin T in CB and OAB-N-3 structures at 14 and 21 days of cell culture. All data are presented as mean ± SD. The *p* values were calculated by student’s *t*-test (*n* = 5/group; *p < 0.05; **p < 0.01; ***p < 0.001). Scale bar, 500 µm (**a**—surface, **e**, **f**—top panel); 200 µm (**a**—cross-section, **b**); 100 µm (**e**, **f**—bottom panel).

By adopting the following printing parameters: UV dose = 120 mJ/cm^2^, temperature = 10 °C, printing pressure = 120 ± 10 kPa, nozzle moving speed = 1 mm/s, cell-laden Gelma filaments (5 wt% Gelma, C2C12 density of 1 × 10^7^ cells/mL) were fabricated using the CB and OAB-N-3 (nozzle shape: N-3) processes. As illustrated in Fig. 6a, the surface and cross-sectional optical images of the cell-laden Gelma struts exhibit round and star shapes for the struts processed with CB and OAB-N-3, respectively. The live (green)/dead (red) images and cell-viability (CB = 92.5 ± 2.2% and OAB-N-3 = 91.9 ± 1.8%) of the struts at 1 and 3 days of cell culture revealed that the processes, including each photo-crosslinking procedure, were safe for the laden cells (Fig. 6b, c).

In addition, we measured cell proliferation using the MTT assay for the struts and discovered that the proliferation rate was not significantly different between the struts. To observe the cell morphology of the Gelma struts, DAPI (blue)/phalloidin (red) images were captured after 14 days of cell culture. In the recorded images, uniaxially aligned F-actin was observed in the Gelma strut with a grooved surface pattern fabricated using the OAB-N-3 process, whereas in the strut printed with the CB process, a similar uniaxial alignment of F-actin was not achieved (Fig. 6e). Furthermore, DAPI/MHC (green) images at 21 days of cell culture exhibited well-aligned and accelerated MHCs, determined by the orientation factor (=[90 − φ]/90, where *φ* = full width at half maximum in an alignment distribution), and more positive index/fusion index values were observed in the Gelma struts processed with OAB-N-3 than in the struts processed with CB (Fig. 6f, g).

To observe the myogenic differentiation of the printed Gelma struts, myogenic gene expression (MHC and troponin T) was observed at 14 and 21 days of cell culture (Fig. 6h). The myogenic genes were upregulated in the Gelma group processed with OAB-N-3; this was owing to the topographical cue (groove shape) inside the nozzle, which was specially designed to induce cell alignment. These results are in good agreement with the results of several studies that have demonstrated that various scaffolds with uniaxial topographical patterns can adjust the alignment of myoblasts and even myotube formation^33–35^. Herein, immortalized C2C12 cells were used to evaluate the in vitro cellular activities. However, the in vitro results may differ when using primary autologous or allogenic cells.

### In vivo studies

**Fig. 7:**
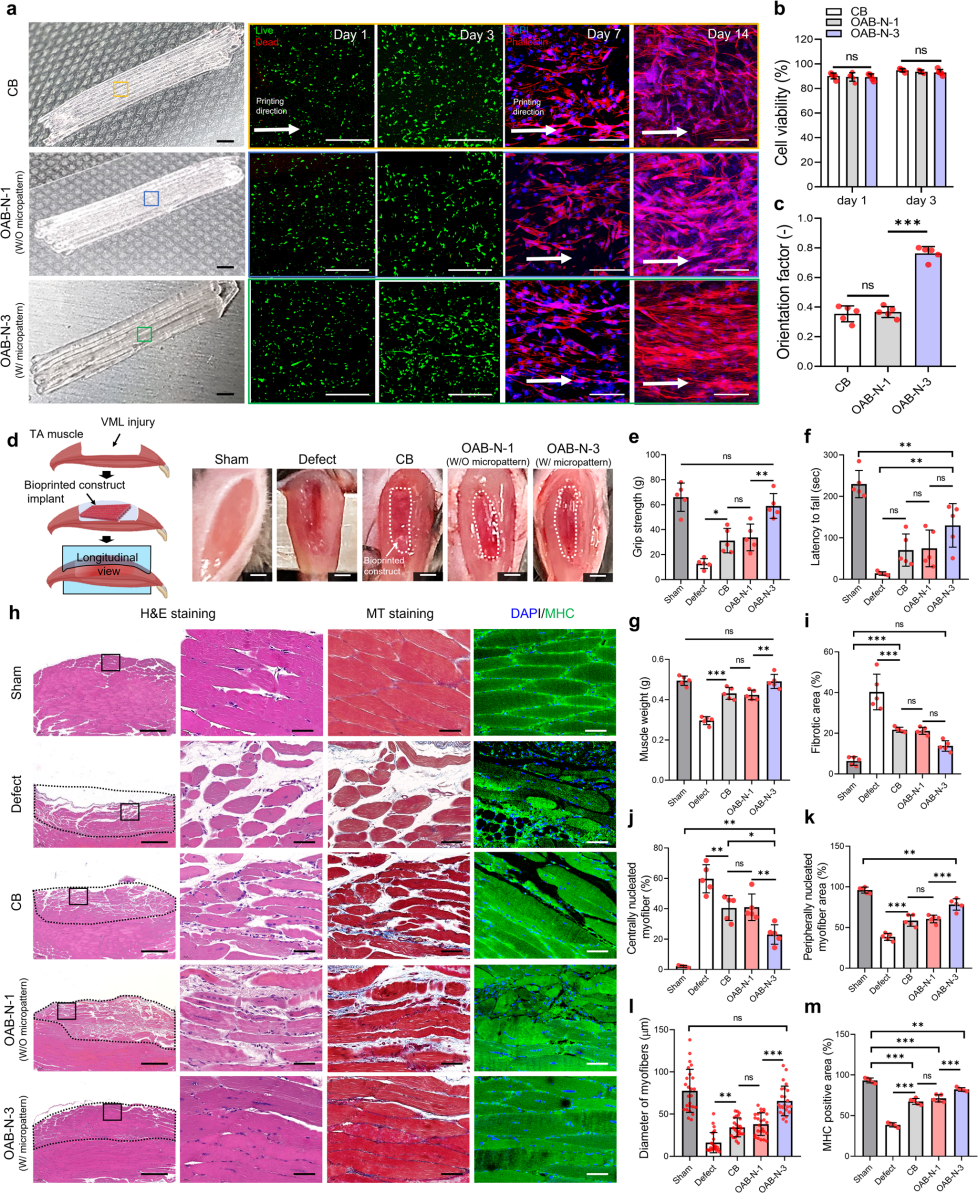
In vivo study. **a** Optical images, live/dead at day 1 and 3, and DAPI/phalloidin images at 7 and 14 days of CB and OAB muscle constructs (OAB-N-1 and OAB-N-3) laden with human adipose stem cells processed with N-1 and N-3 nozzles, respectively. **b** Cell-viability of the muscle constructs at 1 and 3 days of cell culture (*n* = 5 per group, four random fields in each sample) and (**c**) orientation factor using F-actin at 14 days of cell culture (*n* = 5 per group, four random fields in each sample). **d** Gross appearance of the constructs implanted in the TA muscle defect. Quantified (**e**) grip strength, (**f**) latency to fall test, and (**g**) muscle weight after 4 weeks implantation (*n* = 5 per animals, three repeated measurements per sample). **h** H&E (purple: nuclei, pink: cytoplasm), MT (red: muscle fiber, blue: collagen), and DAPI/MHC (green) of transplanted sites, (**i**) fibrotic area (%) (*n* = 5 per group, four random fields in each sample), (**j**) centrally nucleated myofiber (%) (*n* = 5 per group, four random fields in each sample), (**k**) peripherally nucleated myofiber area (%) (*n* = 5 per group, four random fields in each sample), (**l**) diameter of myofibers (μm) (*n* = 25 per group, four random fields in each sample), and (**m**) MHC-positive area (%) (*n* = 5 per group, four random fields in each sample) for sham, defect only, CB, OAB-N-1, and OAB-N-3 samples. All data are presented as mean ± SD. The *p* values were calculated by student’s *t*-test (**b**, **c**), and one-way ANOVA with Tukey’s post hoc test (**e**–**m**) (NS statistical nonsignificance, *p < 0.05; **p < 0.01; ***p < 0.001). Scale bar, 2 mm (**a**—optical, **d**); 500 µm (**a**—live/dead, **h**–H&E); 100 µm (**a**—DAPI/phalloidin); 50 µm (**h**—H&E magnified, MT, DAPI/MHC).

The hASC-laden Gelma struts fabricated using the CB, OAB-N-1 (nozzle shape: N-1), and OAB-N-3 (nozzle shape: N-3) processes were applied to VML tissue regeneration, which was obtained by excising 40% of an original TA muscle in a mouse model^36^. In this analysis, we used hASCs to support the regeneration of defective VML^37,38^ because the differentiation of hASCs into several cell lineages, including a myogenic lineage, can be induced by various chemical and physical stimuli^39^. As illustrated in Fig. 7a, the fabricated hASCs-laden Gelma constructs (cell density = 1 × 10^7^ cells/mL, size = 2 × 4 × 1.6 mm^3^) (CB, OAB-N-1, and OAB-N-3) were applied in the defected muscle region. As expected, the hASCs were alive in all three structures, and the cells laden in the OAB-N-3 group were well aligned in the printing direction compared with those in CB and OAB-N-1 (Fig. 7a–c).

To assess the efficiency of VML muscle regeneration, five groups: (1) sham (no defect), (2) only defect, (3) CB, (4) OAB-N-1, and (5) OAB-N-3 group were implanted in the mouse model (Fig. 7d). Evaluation of grip strength and latency to fall is a reliable assessment of muscle regenerative efficacy^14,40–42^. As such, the results of the grip strength test and the fall latency test in mice 4 weeks after implantation of the constructs are shown in Fig. 7e–g. The OAB-N-3 group showed a significantly higher grip strength than the other groups, and similar grip strength to the sham (Fig. 7e). However, the fall latency time showed a relatively longer latency to fall compared to the other groups but was significantly lower than that of the sham group (Fig. 7f). We attribute these findings to the denervation of the muscle fibers after the VML defect, resulting in functional impairment^42–44^. To assess this effect, the muscle sections harvested were stained with acetylcholine receptors (AchR) (Supplementary Fig. 8a). Significantly higher expressions of AchR were observed in the mice that received OAB-N-3 compared to other groups (Supplementary Fig. 8b). In addition, the muscle weight of the OAB-N-3 group was very similar to that of the sham group (Fig. 7g). Figure 7h illustrates the longitudinal histological results of H&E, MT staining, and DAPI/MHC, and Supplementary Fig. 9 shows the cross-sectional H&E staining. The fibrotic areas, centrally nucleated myofiber, peripherally nucleated myofiber area, diameter of myofibers, and MHC-positive area were measured, and the results are presented in Fig. 7i–m. As illustrated in the results, more efficient muscle regeneration and a low degree of fibrosis (blue area in MT staining) were detected in the OAB-N-3 group compared with the corresponding values in the CB and OAB-N-1 groups, demonstrating that the topographical cues, caused by the groove of the nozzle used in the OAB-N-3 process. Furthermore, the centrally nucleated muscle fibers on the muscle sections harvested from the mice that were processed with OAB-N-3 were considerably lower compared to those of defect, CB, and OAB-N-1^42,45,46^. Based on these results, we carefully predict that OAB-N-3 bioconstruct can induce more effective muscle regeneration than that in the CB and OAB-N-1 groups without any topographical cues.

**Fig. 8:**
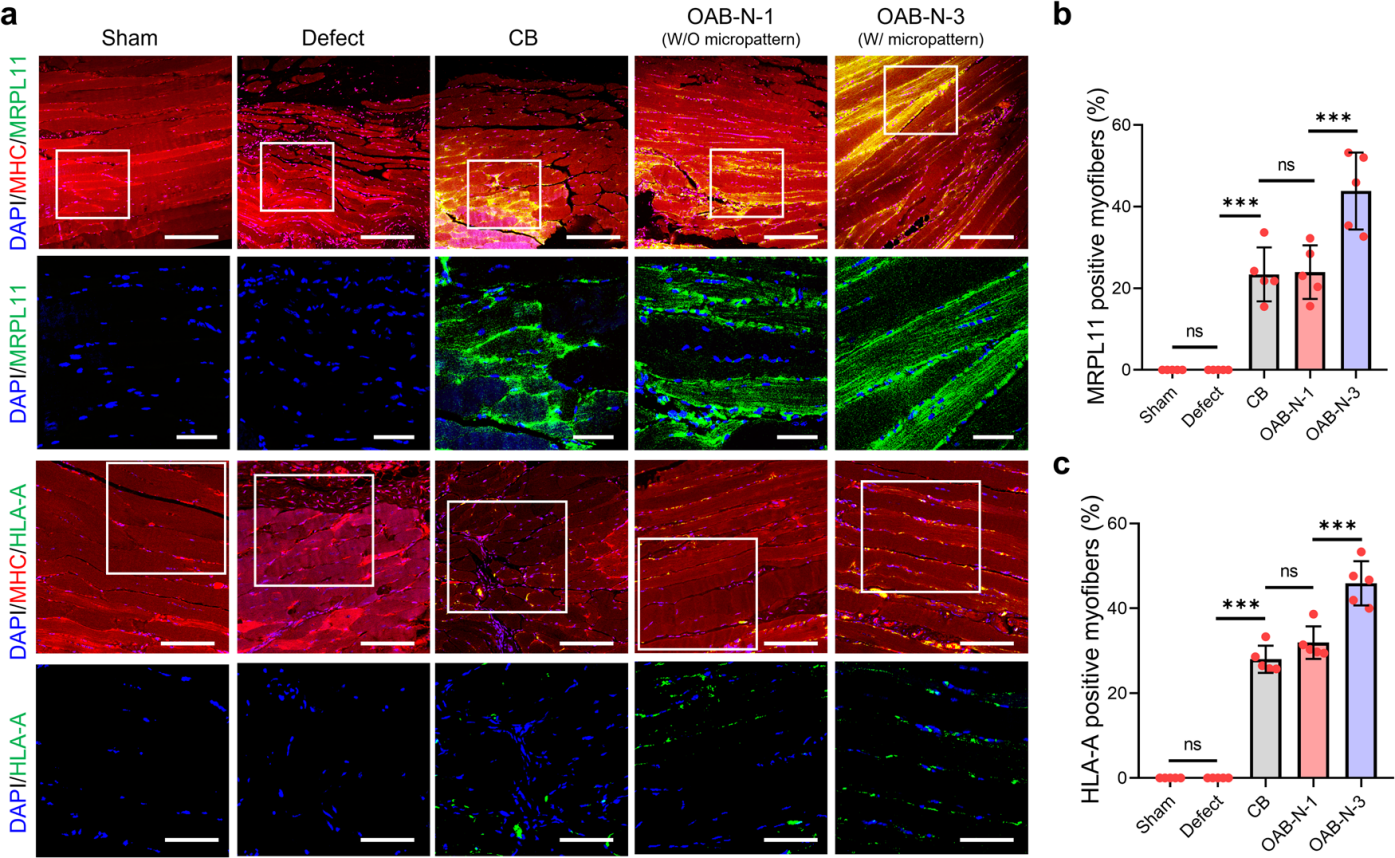
Immunofluorescence images. **a** Double-immunofluorescence images of DAPI (blue)/MHC (red)/MRPL 11 (green) and DAPI (blue)/MHC (red)/HLA-A (green). Quantified (**b**) MRPL11 and (**c**) HLA-A positive myofiber (%) (*n* = 5 per group, four random fields in each sample). All data are presented as mean ± SD. The *p* values were calculated by one-way ANOVA with Tukey’s post hoc test (NS = statistical nonsignificance, *p < 0.05; **p < 0.01; ***p < 0.001). Scale bar, 200 µm (**a**—DAPI/MHC/MRPL11); 50 µm (**a**—DAPI/MRPL11, DAPI/HLA-A); 100 µm (**a**—DAPI/MHC/HLA-A).

More interestingly, myofibers formed in the implanted constructs were originated from the printed hASCs in the CB, OAB-N-1, and OAB-N-3, respectively, as confirmed by double-immunofluorescence for MHC/MRPL11 and MHC/HLA-A at 4 weeks after implantation (Fig. 8a). In particular, it can be observed that the OAB-N-3 group has significantly higher MRPL11 and HLA-A positive muscle fibers than other groups (CB and OAB-N-1) (Fig. 8b, c). Based on the histological results, we can conclude that muscle regeneration using the cell-laden Gelma construct processed using the OAB-N-3 process can be accelerated compared with that using the Gelma construct processed using the CB and OAB-N-1 process. However, despite these favorable muscle regenerative results regarding the proposed construct (OAB-N-3), it is worth pointing out that owing to the small defect sizes of the mouse VML model, the clinical relevance is lacking.

In this study, we developed a novel bioprinting process supplemented with an optical fiber-assisted photo-crosslinking method to fabricate cell-laden filaments with micropatterned surfaces for tissue engineering applications. To obtain biofunctional cell-laden struts with a topographical cue, appropriate printing parameters, such as UV dose and printing temperature, were carefully selected. To demonstrate the feasibility of the bioprinting process, C2C12 myoblasts and hASCs were used to observe in vitro myogenic activities and in vivo volumetric muscle defects, respectively, in a mouse model. As a result of the biomimetic phenotype on the cell-laden Gelma constructs processed with OAB-N-3, improved in vivo VML repair was observed compared to bioconstructs printed via the conventional printing processes. Based on these results, we believe that the newly designed bioprinting process combined with optical fiber-assisted crosslinking can serve as a promising approach for fabricating biofunctional cell-laden constructs for application in various tissue regenerations.

## Methods

### Design of an optic-fiber-assisted nozzle system

The optic-fiber-assisted bioprinting system was composed of three main parts: a 3D printer (DTR3-2210 T-SG; DASA Robot, Korea), an ultraviolet (UV) generator with a UV-spot head (LGA-20562; Liim Tech, Korea), and a nozzle combined with an optical fiber, as presented in Fig. 1a–c. Silica optical fibers (diameter = 200 μm, length = 5.5 cm, numerical aperture = 0.22, Edmund Optics, NJ, USA) with low attenuation to UV radiation (40 db/km) were purchased from Edmund Optics (Barrington, NJ, USA). Plastic nozzles were fabricated using a digital light-processing 3D printer (X-CUBE3, Foshan Stek Technology, China) with photocurable acrylate resin (3DV-301; WOW 3D, South Korea). After printing, the nozzles were washed with ethanol and dried at room temperature. Subsequently, the optical fiber was inserted into the plastic nozzles.

### Preparation of Gelma

Methacrylation of gelatin was performed as previously reported^47^. Briefly, gelatin (10 wt%; 300 g Bloom; Sigma-Aldrich) obtained from porcine skin was dissolved in phosphate-buffered saline solution (PBS). Methacrylic anhydride (Sigma-Aldrich, USA) was added dropwise at 50 °C, and the solution was stirred at 500 rpm for 3 h. Gelma was dialyzed in distilled water using a 12 kDa molecular weight cut off dialysis membrane (Thermo-Fisher Scientific, USA) at 40 °C for 7 days to eliminate the remaining methacrylic anhydride. Finally, Gelma was lyophilized and stored in a deep freezer.

### Gelma bioink

The Gelma solution (5 wt%) was dissolved in PBS containing lithium phenyl-2,4,6-trimethylbenzoylphosphinate (0.05 wt%; Sigma-Aldrich, USA) at 37 °C. To sterilize the Gelma hydrogels, 0.22 μm syringe filters (Thermo-Fisher Scientific, USA) were used. Two different Gelma bioinks were obtained by mixing them with C2C12 mouse myoblasts (CRL-1772; ATCC, Manassas, VA, USA) and hASCs (PT-5006; Lonza, Basel, Switzerland) at a density of 1 × 10^7^ cells/mL.

A Bohlin Gemini HR Nano rotational rheometer (Malvern Instruments, UK) equipped with an acrylic parallel-plate geometry (diameter = 40 mm and gap = 200 μm) was used to evaluate the rheological properties of the Gelma bioinks. A time-sweep test (strain = 1%; temperature = 10 °C; and frequency = 1 Hz) of the Gelma bioink was conducted under various UV conditions. A temperature sweep test (4–45 °C) of the Gelma solution was conducted at a ramping speed (5 °C/min) under a fixed frequency of 1 Hz and a strain of 1%.

### Fabrication of the cell-laden Gelma construct

An optical fiber nozzle, coupled with a three-axis printing system (DTR3-2210 T-SG; DASA Robot, Korea) and dispensing system (AD-3000C; Ugin-tech, Korea), was used to fabricate the cell-laden constructs. The process parameters were as follows: nozzle moving speed = 2 mm/s, bioink flow rate = 0.045 mL/min, barrel temperature = 10 °C and water jacket temperature = 4 °C. During the extrusion process, the bioink was simultaneously exposed to a UV dose [(dose = I_v_ × t; I_v_ = UV intensity (20 mW/cm^2^), t = UV exposure time (6 s)] of 120 mJ/cm^2^ for crosslinking. The UV intensity was measured using a UV light meter (LS125; Linshang, China).

### Cell-laden Gelma construct characterization

Morphological characterization was performed using an optical microscope (BX FM-32; Olympus, Tokyo, Japan) equipped with a digital camera and a scanning electron microscope (SNE-3000M; SEC, Inc., South Korea). The ImageJ software (National Institutes of Health, Bethesda, MD, USA) was used to detect differences between cross-sectional gray values, circularity (=4π × A/P^2^, where A = area and P = perimeter), and aspect ratio determined by the longest length/shortest length. The biodegradability rate was assessed by immersing the samples in PBS at 37 °C for 7 days. Differences in the strut diameter changes were considered to measure the rate of degradation.

Mechanical analyses, including tensile and lap-shear tests of the structures, were performed using a universal tensile machine (Toptech 2000; Chemilab, Korea). To measure the tensile properties, the samples (10 × 3 × 1.6 mm^3^) were tested at speed of 0.5 mm/s using a 30 kgf load cell. To calculate the adhesion properties between the printed constructs (10 mm × 25 × 1.6 mm^3^), the shear test was based on ASTM F2255-05^48^ with a stretch speed of 0.5 mm/s.

### In vitro evaluation of the cell-laden Gelma construct

For in vitro evaluation, cell-laden constructs (C2C12 myoblasts or hASCs) with cell densities of 1 × 10^7^ cells/mL and dimensions of 6 × 30 × 1.6 mm^3^ were bioprinted. The C2C12-laden constructs were cultured in six-well tissue culture plates with Dulbecco’s modified eagle medium (DMEM) high-glucose containing 1% penicillin-streptomycin (Pen-Strep, Thermo-Fisher Scientific, USA) and 10% fetal bovine serum (FBS, Gemini Bio-Products, USA). In addition, we used DMEM high-glucose supplemented with 2% horse serum (Sigma-Aldrich St Louis, USA) to induce myogenesis. The hASC-laden constructs were cultured in six-well tissue culture plates with DMEM low-glucose containing 1% Pen-Strep and 10% FBS. In addition, we used a DMEM low-glucose supplement containing 5% horse serum, 0.1 μM dexamethasone, and 50 μM hydrocortisone. The medium for all samples was changed every 3 days.

Cell viability within the cell-laden constructs was investigated using a live/dead assay kit (Invitrogen, Carlsbad, CA, USA) according to the manufacturer’s instructions. The cell-laden constructs were collected after 1 day of culture, washed with Dulbecco’s phosphate-buffered saline (DPBS) and incubated in a reagent solution (0.15 mM/mL calcein AM and 2 mM ethidium homodimer-1 in DPBS) at room temperature for 40 min for the live/dead assay. Three images of the stained samples were captured using a confocal microscope (LSM 700; Carl Zeiss, Germany) after gentle washing with DPBS. Using these images, we quantified the mean value of cell viability (%) as the ratio of live cells to the total cells by counting the number of live cells (green) and dead cells (red) using the ImageJ software.

Cellular metabolic activity within the construct was measured using a 3-[4,5-dimethylthiazol-2-yl]-2,5 diphenyl tetrazolium bromide (MTT) assay kit (Boehringer Mannheim, Mannheim, Germany). After 1, 3, and 7 days of culture, the activity of mitochondrial dehydrogenase in living cells was measured at an absorbance of 570 nm after exposure to 0.5 mg/mL of the MTT solution at 37 °C for 4 h. Absorbance values obtained from cell-free constructs were subtracted from those obtained from the cell-ladened constructs.

The bioprinted constructs containing C2C12 myoblasts and hASCs were fixed in 3.7% of paraformaldehyde (Sigma-Aldrich) for 30 min, blocked using a serum-free protein blocking agent (Agilent) for 1 h, and permeabilized with 0.1% Triton X-100 (Sigma-Aldrich) for 3 min. The cytoskeleton of cells in the bioprinted constructs was visualized by immunofluorescence using Alexa Fluor 594-conjugated phalloidin (red; 1:100 in DPBS; Invitrogen), and nuclei were stained with diamidino-2-phenylinodole (DAPI) (blue; 1:100 in DPBS; Invitrogen). The orientation factor [=(90° − φ)/90°, φ = full width at half maximum (FWHM)] was analyzed using the ImageJ software.

For myosin heavy chain (MHC) immunofluorescence, the bioprinted constructs were treated with an anti-MF20 primary antibody (5 μg/ml; Developmental Studies Hybridoma Bank, Iowa City, IA, USA) and incubated overnight at 4 °C. The samples were then washed with DPBS and incubated with Alexa Fluor 488-conjugated secondary antibodies (green; 1:50 in DPBS; Invitrogen) for 1 h. Finally, the nuclei were stained with DAPI. MHC/DAPI immunofluorescent images were visualized and captured using a confocal microscope, and myofiber maturity (MHC-positive index and fusion index) was quantified using the ImageJ software.

For real-time quantitative reverse transcription-polymerase chain reaction (qRT-PCR) analysis, the bioprinted constructs were collected after 14 and 21 days of culture, and MHC and troponin T myogenic gene markers were used. Briefly, the cell-laden constructs were treated with a TRIzol reagent (Sigma-Aldrich) to isolate the RNA. The RNA concentration and purity were measured using a spectrophotometer (FLX800T; BioTeK, USA). The RNA samples were treated with RNase-free DNase before cDNA synthesis. cDNA samples were synthesized using the Power SYBRVR Green qPCR Master Mix (Qiagen). A StepOnePlus real-time PCR system (Applied Biosystems, USA) was used to conduct RT-PCR analysis, according to the manufacturer’s protocol. The housekeeping gene glyceraldehyde 3-phosphate dehydrogenase (GAPDH) was used for normalization. The specific primer sequences for the target genes are listed in Table 1.

**Table 1:** Specific primer sequences used for qRT-PCR.

Gene	Host	Primer sequence	
*GAPDH*	Mouse	Forward	5-ATG TTT GTG ATG GGT GTG AA-3
		Reverse	5-ATG CCA AAG TTG TCA TGG AT-3
*MHC*	Mouse	Forward	5-AGC AGA CGG AGA GGA GCA GGA AG-3
		Reverse	5-CTT CAG CTC CTC CGC CAT CAT G-3
*TnT*	Mouse	Forward	5′-TCA ATG TGC TCT ACA ACC GCA-3′
		Reverse	5′-ACC CTT CCC AGC CCC C-3′

*GAPDH* Glyceraldehydes-3-phosphate dehydrogenase, *MHC* Myosin heavy chain, *TnT* Troponin T.

### Mouse tibialis anterior (TA) muscle defect injury model

TA muscle defects were created in C57BL/6 mice (male, 10 weeks old, DooYeol Biotech, Inc., Seoul, Korea), and all animal procedures were performed according to the protocol approved by the Institutional Animal Care and Research Advisory Committee at the Sungkyunkwan University School of Medicine Laboratory Animal Research Center and complied with the regulations of the Institutional Ethics Committee (SKKUIACUC2021-08-11-2). Briefly, mice were randomly divided in to five groups (total of 25 mice, *n* = 5 per group). Prior to implantation, the mice were first anesthetized using 3% v/v isoflurane, and their muscles were isolated from the fascia by incision of the skin under the left and right legs. Following this, the extensor digitorum longus and extensor hallucis longus were removed to induce volumetric muscle loss (VML) injury, and approximately 40% of the TA muscles were excised and weighed (Supplementary Table 1). For VML restoration, hASC-laden bioprinted constructs (2 × 4 × 1.6 mm^3^) were transplanted into TA muscle defect regions and covered with sutured fascia and skin. After implantation, the mice received a subcutaneous injection of 0.5 mg/kg buprenorphine to control pain and were given food and water normally after the operation. After 4 weeks of implantation, the mice were euthanized *via* CO_2_ inhalation and secondary cervical dislocation. Before transplantation, the hASC-laden constructs were cultured in a growth medium for 1 day to stabilize the cells^31,49^.

### Muscle functional evaluations

The skeletal muscle functionality of mice that received various treatments was assessed by measuring grip strength and latency to fall. The maximum grip strength of the mice at 4 weeks post-transplantation was measured by pulling the mouse parallel to the grid line and measuring the grip strength (grip strength meter, BIO-G53, BIOSEB, FL, USA)) as the described procedure^50^. Additionally, the latency to fall was evaluated by measuring the time elapsed after the mouse was placed on a rod. The maximum latency period was set at 300 s. Each experiment was repeated thrice per mouse. An interval duration of 5 min was provided between the repetitions (*n* = 5 per animals, three repeated measurements per sample).

### Histological and immunofluorescence staining

For histological staining, the harvested TA muscle samples were fixed with 10% neutral-buffered formalin for 24 h. Then, the samples were dehydrated, paraffin-embedded, and sectioned into five-micron-thick slices. The muscle sections were stained with hematoxylin and eosin (H&E) and *Masson’s trichrome (MT). The H&E-stained images and MT-stained images were analyzed by ImageJ software to quantify the centrally nucleated myofibers*, peripherally nucleated myofiber area *and the fibrotic area respectively (n* = *5 per sample, four random fields in each sample)*. The centrally nucleated myofibers and peripherally nucleated muscle fibers are manually counted using the multipoint tool in imageJ. The centrally nucleated myofiber was obtained by dividing the number of centrally nucleated myofibers to that of the total number of muscle fiber. In addition, the peripherally nucleated myofiber area was calculated via dividing the area of peripherally nucleated myofibers to that of the total muscle fiber area.

For immunofluorescence staining, the sectioned samples were treated with an anti-MF20 primary antibody (5 μg/ml; Developmental Studies Hybridoma Bank, Iowa City, IA), anti-MRPL11 (rabbit polyclonal antimitochondrial ribosomal protein L11, species reactivity: human, 1:100 dilution; Abcam, Cambridge, UK), anti-HLA-A (species reactivity: human, 1:100 dilution; Abcam, Cambridge, UK), anti- CHRNB2 (neuronal acetylcholine receptor subunit beta-2, 1:100 dilution, Thermo-Fisher Scientific, USA) and incubated overnight at 4 °C. The samples were then washed with DPBS and incubated with Alexa Fluor 488-conjugated secondary antibodies (green; 1:50 in DPBS; Invitrogen) or Alexa 594-conjugated secondary antibodies (red; 1:50 in DPBS; Invitrogen) and Alexa 488-conjugated anti-rabbit IgG (1:200 dilution, Invitrogen) for 1 h. Finally, the nuclei were stained with DAPI. MHC/DAPI immunofluorescence images were captured using a confocal microscope. The area of muscle fiber per field (%) (*n* = 5 per sample, four random fields in each sample), diameter of myofibers (*n* = 25 per sample, four random fields in each sample), and MHC-positive area (%) (*n* = 5 per sample, four random fields in each sample) were measured with immunofluorescent images for MHC/DAPI (×400 magnification) in a blinded fashion. Area of MRPL11 and HLA-A positive myofibers (%) were measured with double-immunofluorescent images for MHC/MRPL11 and MHC/HLA-A (×400 magnification) in a blinded fashion (*n* = 5 per sample, four random fields in each sample). All sample images were quantified using the ImageJ software. All values are presented as mean ± standard deviation.

### Statistical analyses

SPSS software (SPSS, Inc., USA) was used to perform the statistical analyses. Comparisons of two groups were evaluated by *t*-test, and three or more groups were compared by assessing one-way or two-way analysis of variance (ANOVA) with Tukey’s HSD post hoc test. Values of *p < 0.05, **p < 0.01 and ***p < 0.001 were considered statistically significant.

### Reporting summary

Further information on research design is available in the Nature Research Reporting Summary linked to this article.

## Supplementary information

Supplemental Information

Reporting Summary

## Data Availability

The data that support the findings of this study are available from the corresponding author upon reasonable request.
