# Supplementary material for: 3D bioprinting using a new photo-crosslinking method for muscle tissue restoration
Source: NPJ Regen Med. 2023 Mar 31;8:18. doi: 10.1038/s41536-023-00292-5 (PMC10066283; doi:10.1038/s41536-023-00292-5)
Supplement: Supplementary file 1 — Supplemental Information [file 41536_2023_292_MOESM1_ESM.pdf]

Supplementary Table 1. The weight of the total and removed 40% of tibialis anterior (TA) muscle in C57BL/6 mouse.

Number of mice	TA muscle weight (g)	Removed 40% of the TA muscle weight (g)
1	0.443	0.1772
2	0.44	0.176
3	0.451	0.1844
4	0.451	0.1804
5	0.453	0.1812
6	0.456	0.1824
7	0.445	0.178
8	0.444	0.1776
9	0.453	0.1812
10	0.45	0.18
Mean ± SD	0.4486 ± 0.005	0.1798 ± 0.003

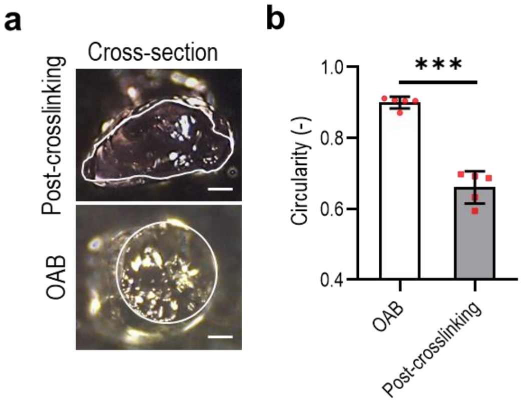

Supplementary Figure 1. Comparison of circularity values between struts fabricated using OAB and post-crosslinking method. a Cross-sectional optical images and **b** measured circularity values of struts fabricated using OAB and post-crosslinking method. All data are presented as mean ± SD. The p-values were calculated by student's t-test ($n = 5$ per group; NS = statistical nonsignificance, $^{*}p < 0.05$, $^{**}p < 0.01$ and $^{***}p < 0.001$). Scale bar, 200 μm (a).

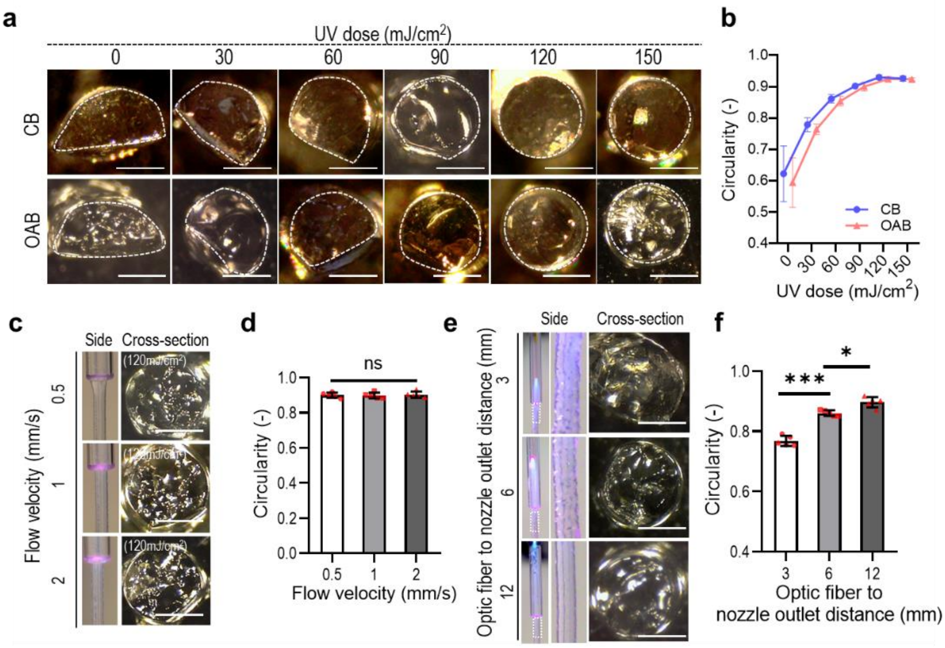

Supplementary Figure 2. Optimization of OAB-based 3D printing of struts by crosslinking method, flow velocity, and optic fiber distance. a Cross-sectional optical images and **b** measured circularity values of struts fabricated using OAB and CB crosslinking method with various UV doses (0 ~ 150 mJ/cm^2). **c** Optical images of extruded strut using the OAB method with varying flow velocities and **d** measured circularity values. **e** Optical images of extruded strut using the OAB method with the optic fiber set in various nozzle outlet distances and **f** measured circularity values. All data are presented as mean ± SD. The *p*-values were calculated by one-way ANOVA with Tukey's post-hoc test (n = 5 per group; $^{*}p < 0.05$, $^{**}p < 0.01$ and $^{***}p < 0.001$). Scale bar, 500 μm (a,c,e).

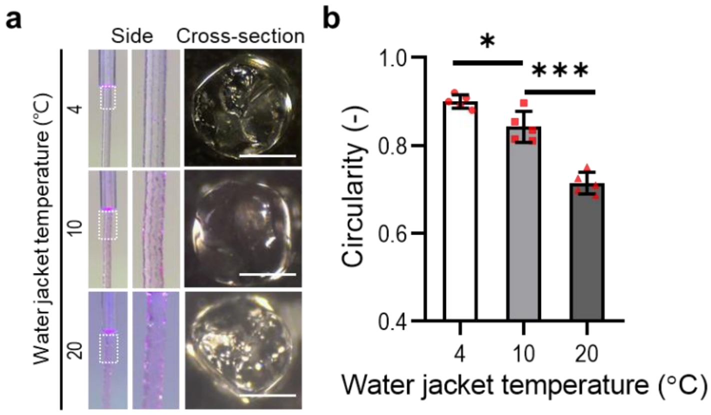

Supplementary Figure 3. Effect of water jacket temperature on the circularity of extruded struts. **a** Optical images and **b** measured circularity values of extruded struts using various water jacket temperatures (4, 10, and 20 °C). All data are presented as mean ± SD. The p-values were calculated by one-way ANOVA with Tukey's post-hoc test (n = 5 per group; $^{*}p < 0.05$, $^{**}p < 0.01$ and $^{***}p < 0.001$). Scale bar, 500 µm (a).

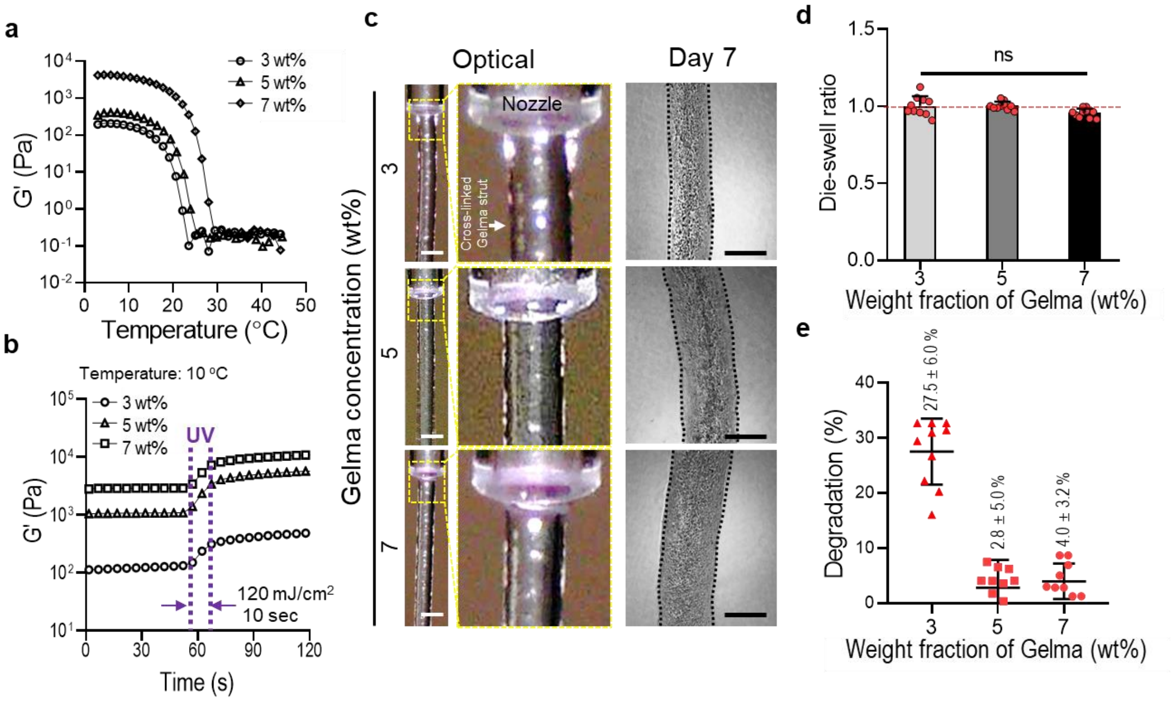

Supplementary Figure 4. Mechanical and degradation properties of 3D-printed Gelma struts with varying concentrations after UV exposure and *in vitro* Culture. Storage moduli of 3, 5, and 7 wt% Gelma bioink obtained via **a** temperature sweep and **b** time sweep under UV exposure (120 mJ/cm^2 and 10 s). **c** Optical images of the printed shape of Gelma struts processed via OAB (UV dose = 120 mJ/cm^2) with various Gelma concentrations, and optical images of the printed struts at 7 days of culture. **d** Die-swelling ratios for various Gelma concentrations. **e** Degradation of printed Gelma struts before and after 7 days of culture. All data are presented as mean ± SD. The *p*-values were calculated by one-way ANOVA with Tukey's post-hoc test (n = 10 per group; $^{*}p < 0.05$, $^{**}p < 0.01$ and $^{***}p < 0.001$). Scale bar, 1 mm (c-strut); 500 μm (c-cultured).

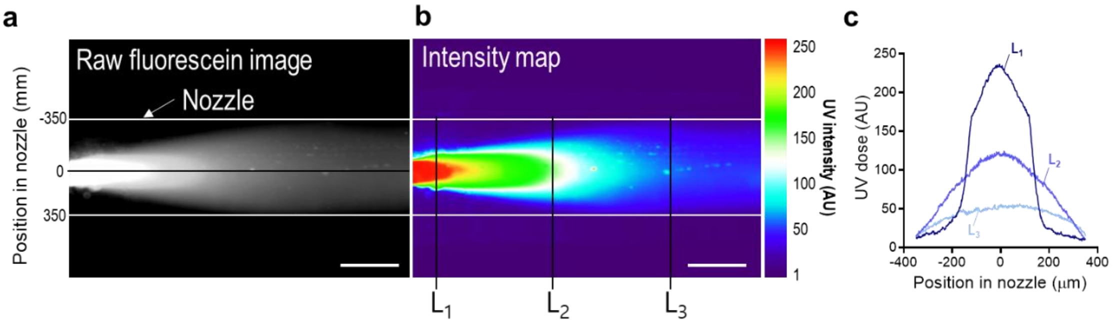

Supplementary Figure 5. Visualization and characterization of UV light emitted from an optic fiber. a Fluorescence image of UV light emitted from optic fiber (visualized using FITC filter) and **b** UV light intensity dispersion image. **c** Distribution of UV light intensity throughout the nozzle geometry. Scale bar, 500 μm (a,b).

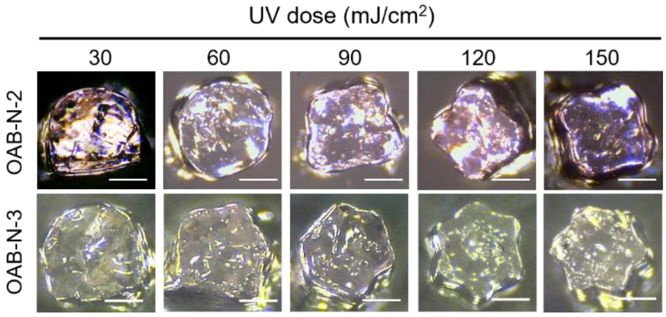

Supplementary Figure 6. Optical images of extruded struts using OAB-N-2 and OAB-N-3 nozzle. Scale bar, 300 μm.

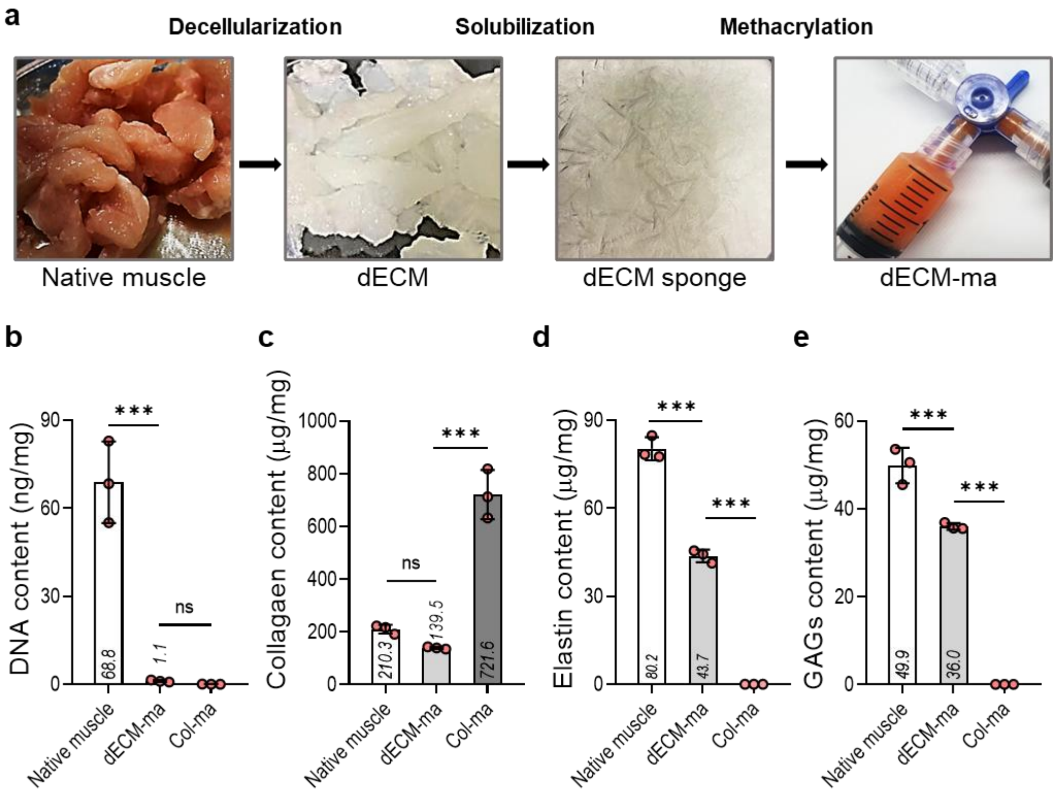

Supplementary Figure 7. Characterization of decellularization/methacrylation process using muscle tissue. a Optical images of the decellularization/methacrylation process using muscle tissue. **b** DNA, **c** collagen, **d** elastic, and **e** GAG contents for the native muscle tissue, dECM-ma and Colma. All data are presented as mean ± SD. The *p*-values were calculated by one-way ANOVA with Tukey's post-hoc test (n = 3 per group; $^{*}p < 0.05$, $^{**}p < 0.01$, and $^{***}p < 0.001$).

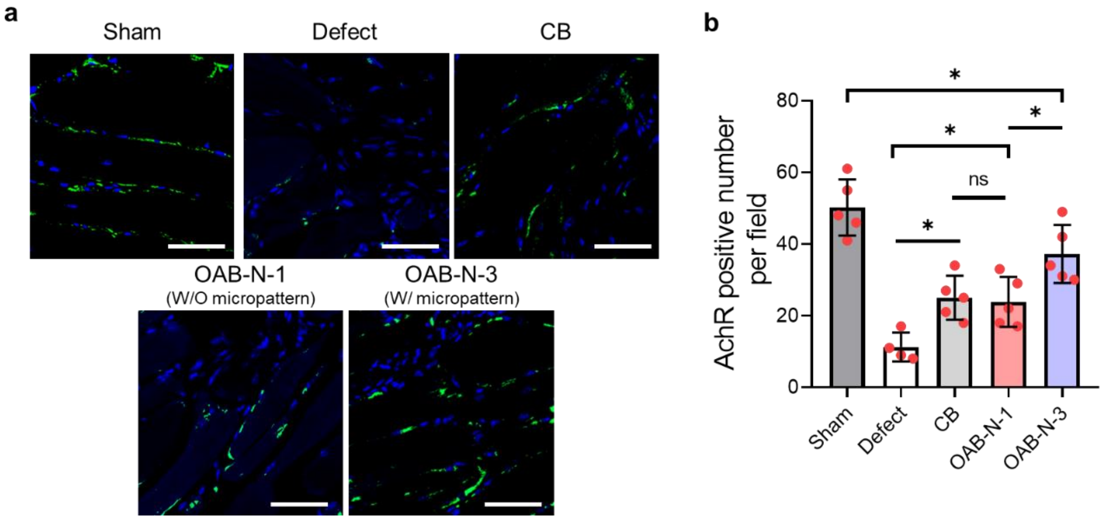

Supplementary Figure 8. Quantification of AchR expression in muscle tissue using immunofluorescence imaging. a Immunofluorescence images of DAPI (blue)/AchR (green) and **b** positive number per field. (*n* = 5 per group). All data are presented as mean ± SD. The *p*-values were calculated by one-way ANOVA with Tukey's *post-hoc* test (NS = statistical nonsignificance, $^{*}p < 0.05$, $^{**}p < 0.01$ and $^{***}p < 0.001$). Scale bar, 50 µm (a).

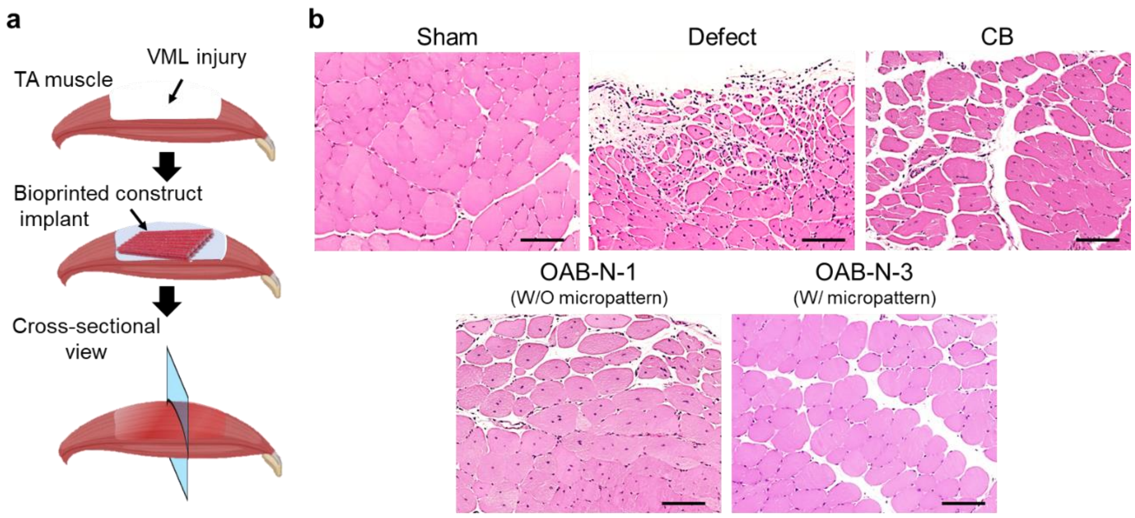

Supplementary Figure 9. Histological image of cross-sectional skeletal muscle tissue. a A schematic diagram of bioconstruct implant on VML defect to TA muscle. **b** Cross-sectional image of H&E (purple: nuclei, pink: cytoplasm) staining. Scale bar, 100 µm (b).
